# Effect of Clodronate on the Pathogenesis in a Mouse Model of Lysosomal Acid Lipase Deficiency

**DOI:** 10.3390/ijms27198694

**Published:** 2026-09-29

**Authors:** Ryuichi Mashima, Shuji Takada, Kei Murayama, Takashi Hamazaki

**Affiliations:** 1 Department of Clinical Laboratory Medicine, National Center for Child Health and Development, 2-10-1 Okura, Setagaya-ku, Tokyo 157-8535, Japan; 2 Department of Systems BioMedicine, National Research Institute for Child Health and Development, 2-10-1 Okura, Setagaya-ku, Tokyo 157-8535, Japan; 3 Diagnostics and Therapeutics of Intractable Diseases, Intractable Disease Research Center, Graduate School of Medicine, Juntendo University, Bunkyo-ku, Tokyo 113-8421, Japan; kmuraya@mri.biglobe.ne.jp; 4 Department of Pediatrics, Graduate School of Medicine, Osaka Metropolitan University, Osaka 545-8585, Japan

**Keywords:** Lysosomal acid lipase deficiency, clodronate, macrophage

## Abstract

Lysosomal acid lipase deficiency (LAL-D, OMIM #309900), caused by mutations of the *LIPA* gene, is involved in two continuum disease phenotypes: Wolman disease in infants and cholesteryl ester storage disease in adults. Their major phenotypes include steatosis, hepatomegaly, splenomegaly, and elevated liver enzymes. Enzyme replacement therapy has been used for the treatment of LAL-D. In this study, we examined the therapeutic effect of liposomal clodronate in an LAL-D mouse model. When liposomal clodronate was administered intraperitoneally by a single administration at 200 mg/kg to adult mice, organ cholesterol and triglyceride levels partially decreased. Furthermore, a significant correction of the relative organ weight of the liver and spleen as well as a reduction in splenic lipids were observed when liposomal clodronate was administered at 50 mg/kg to young mice intraperitoneally by multiple administrations. These results demonstrate that liposomal clodronate might have a therapeutic effect on the LAL-D mouse model.

## 1. Introduction

Lysosomal acid lipase deficiency (LAL-D, OMIM #309900) is caused by a deficiency of enzyme activity of the *LIPA* gene. It is characterized by an accumulation of cholesteryl esters (CEs) and triglycerides (TGs) in the body [1,2,3,4,5]. LAL-D has two disease subtypes with a continuum presentation. Wolman disease is a severe type of LAL-D with typical manifestations of hepatosplenomegaly, diarrhea, vomiting, and severe hepatic disorder. Patients with this condition have a short life expectancy [6,7]. In contrast, cholesteryl ester storage disorder (CESD) is an attenuated disease subtype of LAL-D, with abnormal serum lipids. For Wolman disease, hematopoietic stem cell transplantation has been used as a major therapeutic option [8,9]. More recently, enzyme replacement therapy has been available [8,9,10]. Lipid-lowering agents, such as statins, have also been used for CESD [11]. It is generally accepted that the therapeutic benefit is maximized when treatment begins as early as possible. For this reason, neonatal screening, a social program used to identify affected individuals in the neonatal period, has been studied intensively [12,13,14]. From a diagnostic viewpoint, elevated liver enzymes are a good biomarker in second-tier confirmatory assays for neonatal screening [15].

A murine model for LAL-D has previously been established [16]. This mouse model showed a shortened lifespan with hepatosplenomegaly associated with hepatic accumulation of lipids including TGs and CEs [16]. In the liver, multiple cell types, including hepatocytes, Kupffer cells, hepatic stellate cells, lymphocytes, and endothelial cells, are found in hepatic tissue. Hepatocytes, which comprise the largest population of cells in the liver, play a major role in metabolizing lipids [17,18,19]. The Kupffer cell is a tissue-resident macrophage with the second largest population in the liver. These cells act as scavengers of debris in the liver [20]. Hepatic stellate cells localize underneath blood vessels, and are reportedly involved in the formation of fibrosis [21]. In parallel, this mouse model has been used in the development of enzyme replacement therapy and adeno-associated virus-based gene therapy [22,23]. Supraphysiological expression of the *LIPA* gene shows inflammatory phenotype in mice [24].

Metabolic dysfunction-associated steatotic liver disease (MASLD) and metabolic dysfunction-associated steatohepatitis (MASH) are prevalent in developed countries [25,26]. Usually, this disease develops from a healthy liver and is followed by steatosis and cirrhosis. Its etiology remains unclear, but an excess intake of lipids and carbohydrates is considered a major factor. Recent population-selected screening results indicate that some LAL-D-affected individuals may be found in the population of MASLD and MASH [5,27]. Kuloglu Z et al. screened 810 children with cryptogenic liver disorders involving unexplained transaminase elevation, unexplained hepatomegaly, and obesity-unrelated liver steatosis, and identified two positives based on an enzyme assay using dried blood spots [5]. Using a similar technique, Tibani A et al. reported both Wolman and CESD-affected individuals involving similar cryptogenic liver diseases in a large-scale clinical screening in France [15]. Because LAL-D shows non-specific manifestations widely found in other liver disorders, measurement of enzyme activity using dried blood spot testing is beneficial when identifying affected individuals.

Liposomal clodronate has been used as a macrophage-depleting agent [28,29]. It has proven effective in various sites, including the peritoneum, liver, and spleen [30,31,32]. For therapeutic purposes, the beneficial role of liposomal clodronate in treating diet-induced obesity has been reported [33]. Administration of liposomal clodronate for early-stage obesity has been particularly effective, suggesting that this preventative protocol exhibits a higher efficacy at this stage [34]. In this obesity model, the accumulation of Kupffer cells was significantly attenuated upon clodronate administration, raising the possibility that reduced body weight gain in clodronate-treated animals could be modulated by these cells. Apart from these diet-induced obesity models, the effect of clodronate on steatosis in Lipa-deficient animals has been poorly understood.

In this study, we examined the therapeutic effect of liposomal clodronate on our newly developed LAL-D mouse model with steatosis and hepatosplenomegaly [35]. Removal of splenic lipids was the most noted outcome of liposomal clodronate administration to our LAL-D animal model. We also found a partial normalization of the relative liver weight, while hepatic lipids remained unaltered.

## 2. Results

**Figure 1:**
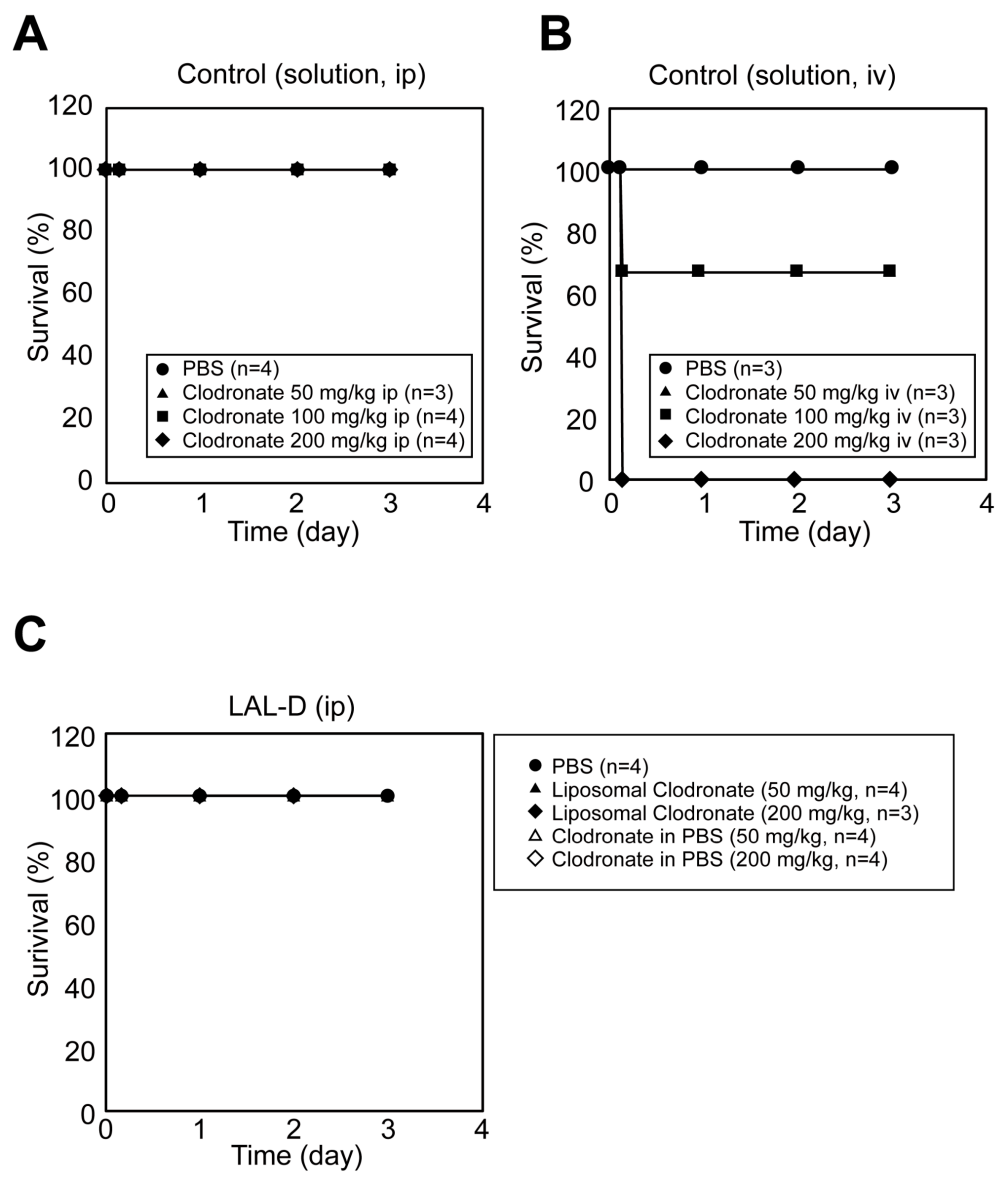
Optimization of clodronate administration route and its formulation. (**A**) Survival of young control mice at 2 weeks of age after administration of clodronate solution intraperitoneally (**A**) and intravenously (**B**). Note that all mice with clodronate at 200 mg/kg intravenously died immediately after administration. (**C**) The survival of young LAL-D mouse model with clodronate solution (clodronate in PBS) or liposomal clodronate. Commercially obtained liposomal clodronate (averaged diameter = 300 μm, Katayama Chemical Industries, Osaka, Japan) was diluted with PBS (Gibco) at the indicated concentration. Clodronate solution in PBS was prepared by an aliquot of aseptical clodronate (Tokyo Chemical Industries, Tokyo, Japan) in PBS.

First, to examine a suitable administration route of clodronate, we compared the survival of control mice at 2 weeks of age receiving clodronate in solution either intraperitoneally or intravenously (Figure 1A,B). Although all mice survived when they received up to 200 mg/kg clodronate intraperitoneally (Figure 1A), those mice receiving 200 mg/kg clodronate intravenously in solution died immediately (Figure 1B). This lethal effect was dose-dependent, because 67% of mice with clodronate 100 and 50 mg/kg iv (i.e., two mice out of three in each group) survived under these experimental conditions (Figure 1B). To examine the effect of the formulation, we administered a solution of clodronate in phosphate buffered-saline (PBS) and a commercially available liposomal clodronate (Figure 1C). Our results showed that the administration of this liposomal clodronate at 200 mg/kg intraperitoneally was tolerated by our LAL-D mouse model. Based on these data, we decided to administer liposomal clodronate at 50–200 mg/kg intraperitoneally in a subsequent study.

**Figure 2:**
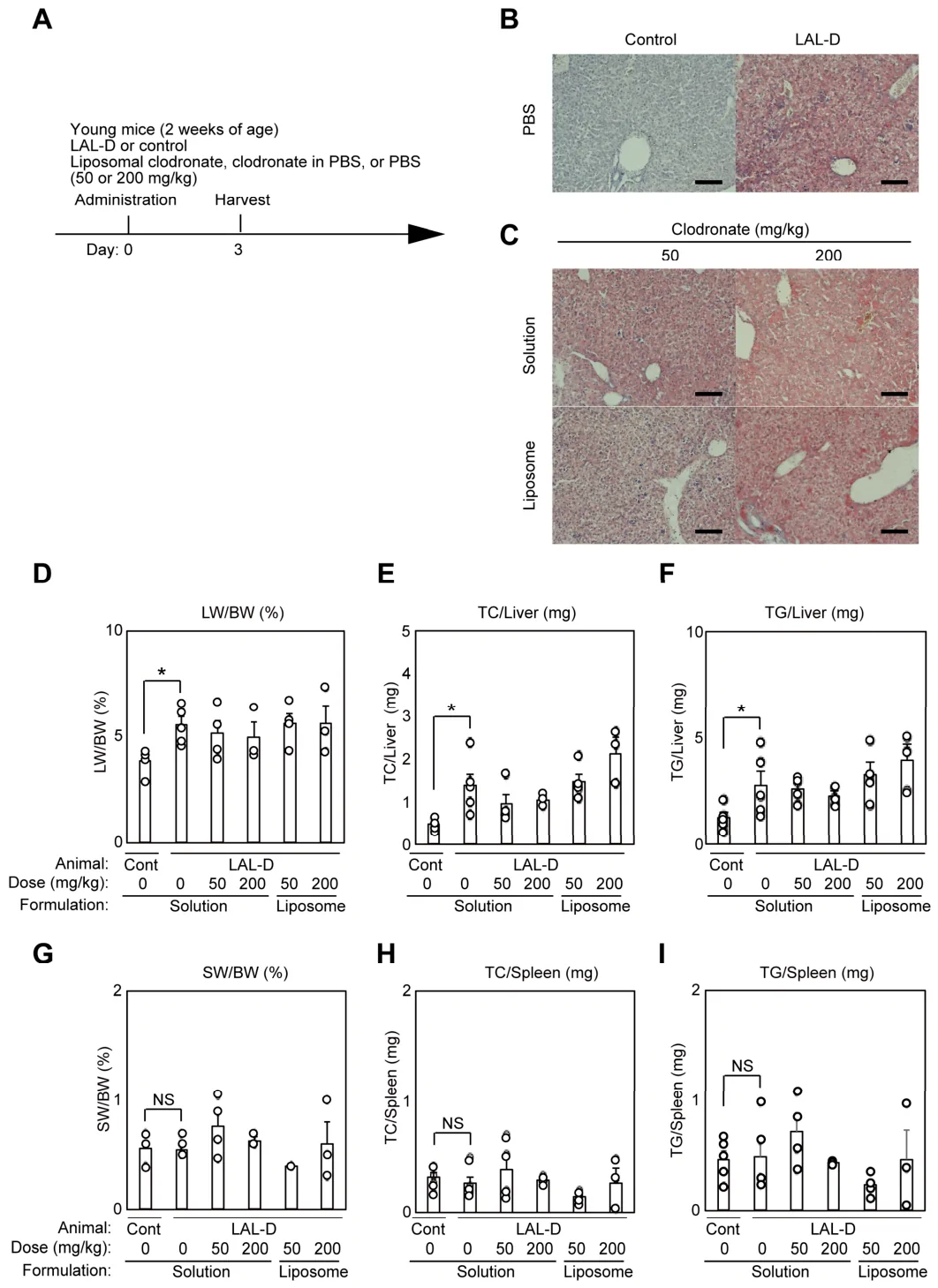
Single-dose administration of clodronate to young LAL-D mouse model at 2 weeks of age. (**A**) Experimental protocol. Young mice at 2 weeks of age received either liposomal clodronate, clodronate in solution in PBS, or PBS for control intraperitoneally. Tissues were harvested for further examination at day 3. (**B**) Oil Red O staining of liver section. Images of control mice with PBS (left, *n* = 4) and LAL-D mice with PBS (right, *n* = 3) are shown. Scale bar: 100 μm. (**C**) Oil Red O staining of liver sections. Images of LAL-D mice with 50 mg/kg clodronate solution in PBS (top left, *n* = 5), LAL-D mice with 200 mg/kg chlodronate in PBS (top right, *n* = 3), LAL-D mice with 50 mg/kg liposomal clodronate (bottom left, *n* = 4), LAL-D mice with 200 mg/kg liposomal clodronate (bottom right, *n* = 3) are shown. (**D**) Changes in liver weight (LW) to body weight (BW) ratio (LW/BW). (**E**) Changes in total cholesterol (TC) in the liver. (**F**) Changes in triglyceride (TG) in the liver. (**G**) Changes in spleen weight (SW) to BW ratio (SW/BW). (**H**) Changes in TC in the spleen. (**I**) Changes in TG in the spleen. An asterisk denotes *p* < 0.05. NS, not significant. Same number of specimens was examined in each group in biochemistry.

To understand the difference in the therapeutic effect of formulation, we treated this young LAL-D mouse model at 2 weeks of age with clodronate administered as a single dose (Figure 2A). In PBS-treated mice, Oil Red O staining was positive in the LAL-D mouse model but not in the control mice (Figure 2B and Figure S1). When we administered clodronate in solution at 50 or 200 mg/kg intraperitoneally, the overall histological appearance remained unaltered on day 3 (Figure 2C, top). Subsequently, when we administered the same amount of clodronate in liposomes, we found that there was almost no therapeutic effect at 50 or 200 mg/kg intraperitoneally (Figure 2C bottom). In this LAL-D mouse model, the liver weight (LW) to body weight (BW) ratio was elevated, demonstrating that hepatomegaly (a hallmark of LAL-D manifestation) was established at 2 weeks of age (Figure 2D). Total cholesterol (TC) and TG were consistently increased in the LAL-D mouse model at this stage (Figure 2E,F). However, the spleen weight (SW) to BW ratio and the levels of TC and TG were unchanged in the spleen (Figure 2G–I). When we administered clodronate in either solution or liposome at 50 or 200 mg/kg intraperitoneally, there were no obvious changes in these biomarkers in 2-week-old mice. At 2 weeks of age, an elevation of TC in the liver was associated with that in liver protein as well (Supplementary Figure S2).

**Figure 3:**
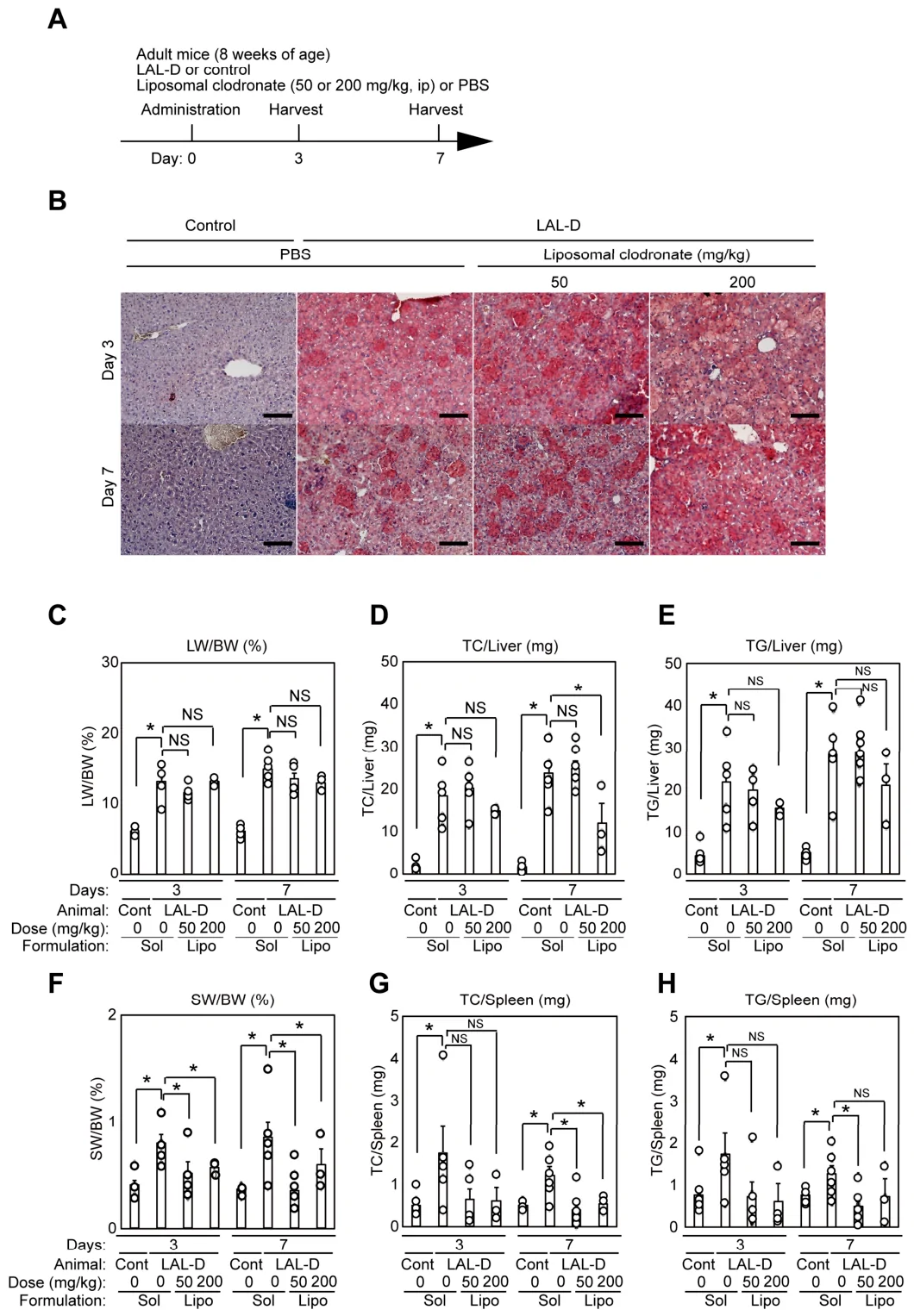
Single-dose administration of clodronate to adult LAL-D mouse model at 8 weeks of age. (**A**) Experimental protocol. LAL-D mice at 8 weeks of age received either 200 or 50 mg/kg liposomal clodronate intraperitoneally for systemic macrophage depletion. As control animals, heterozygote mice were used. LAL-D mice receiving PBS were similarly examined for further comparison. These mice were sacrificed at either day 3 or day 7. (**B**) Oil Red O staining of liver section. Images of control mice with PBS on day 3 (top far left, *n* = 7), LAL-D mice with PBS on day 3 (top middle left, *n* = 4), LAL-D mice with 50 mg/kg liposomal clodronate on day 3 (top middle right, *n* = 5), LAL-D mice with 200 mg/kg liposomal clodronate on day 3 (top far right, *n* = 3), control mice with PBS on day 7 (bottom far left, *n* = 7), LAL-D mice with PBS on day 7 (bottom middle left, *n* = 4), LAL-D mice with 50 mg/kg liposomal clodronate on day 7 (bottom middle right, *n* = 7), LAL-D mice with 200 mg/kg liposomal clodronate (bottom far right, *n* = 3). Scale bar: 100 μm. (**C**) Changes in LW to BW ratio. (**D**) Changes in TC in the liver. (**E**) Changes in TG in the liver. (**F**) Changes in SW to BW ratio. (**G**) Changes in total TC in the spleen. (**H**) Changes in TG in the spleen. An asterisk denotes *p* < 0.05. NS, not significant. Same number of specimens was examined in each group in biochemistry.

Next, we investigated the possible therapeutic effect of clodronate in the adult LAL-D mouse model at 8 weeks of age (Figure 3A). Similar to the young LAL-D mouse model at 2 weeks of age, on day 3, Oil Red O staining was only positive in the LAL-D mouse model, but not in the controls, when these animals were treated with PBS as the control agent (Figure 3B, top far left and center left, Supplementary Figure S3). A similar histology was observed when the LAL-D mouse model was administered liposomal clodronate at 50 mg/kg (Figure 3B, top center right). In contrast, on day 3, we found a disappearance of this strongly stained area with Oil Red O at 200 mg/kg (Figure 3B, top far right). On day 7, such a histological alteration remained unchanged at 50 mg/kg (Figure 3B, bottom center right). However, an accumulation of newly developed clusters of macrophages with deformed shapes emerged at 200 mg/kg (Figure 3B, bottom far right).

Next, we further examined the organ weights and biochemistry of these animals. At 8 weeks of age, the adult LAL-D mouse model showed an elevation of all parameters involving LW/BW, TC, and TG in the liver and SW/BW, TC, and TG in the spleen (Figure 3C–H and Supplementary Figure S4). When the liposomal clodronate was administered to the adult LAL-D mouse model, nearly half of the parameters became normalized to control levels, particularly on day 7. For example, an administration of the liposomal clodronate normalized the splenomegaly of this adult LAL-D mouse model at 50 and 200 mg/kg liposomal clodronate intraperitoneally (Figure 3F). Furthermore, the levels of splenic TC and TG on day 7 normalized to the control level when liposomal clodronate was administered at 50 mg/kg intraperitoneally (Figure 3G,H). A similar trend was observed in splenic TC at 200 mg/kg (Figure 3G). Similarly—but to a lesser extent—the TC in the liver at 200 mg/kg clodronate on day 7 reduced significantly when this experimental condition occurred in an adult LAL-D mouse model at 8 weeks of age (Figure 3C–E). Other biomarkers in the liver appeared to be almost unaffected.

**Figure 4:**
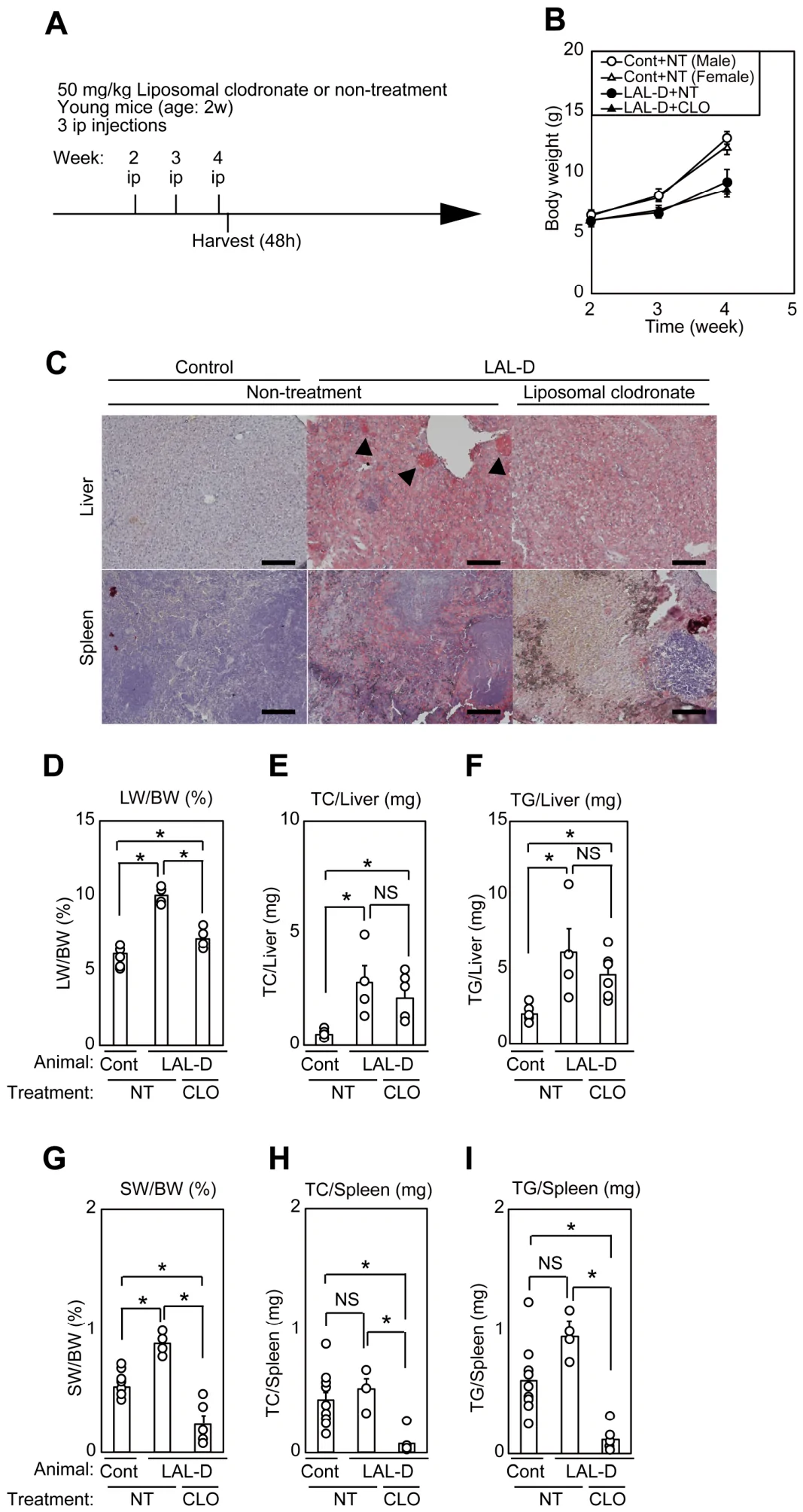
Multi-dose administration of liposomal clodronate to young LAL-D mouse model at 2 weeks of age. (**A**) Experimental protocol. LAL-D mice at 2 weeks of age received liposomal clodronate at 50 mg/kg intraperitoneally (CLO). After clodronate injection at week 4, tissues were harvested at 48 h of final administration. (**B**) Changes in body weight of control and LAL-D mouse model treated with 50 mg/kg liposomal clodronate intraperitoneally. KO, Lipa-deficient; Het, heterozygote; CLO, clodronate; NT, no treatment. (**C**) Oil Red O staining of histology section. Images of control mice with no treatment in the liver (top left, *n* = 4), LAL-D mice with no treatment in the liver (top middle, *n* = 4), LAL-D mice with 50 mg/kg liposomal clodronate in the liver (top right, *n* = 6), control mice with no treatment in the spleen (bottom left, *n* = 4), LAL-D mice with no treatment in the spleen (bottom center, *n* = 3), LAL-D mice with 50 mg/kg liposomal clodronate in the spleen (bottom right, *n* = 4). Arrow heads indicate Kupffer cells. Scale bar: 100 μm. (**D**) Changes in LW to BW ratio. (**E**) Changes in TC in the liver. (**F**) Changes in TG in the liver. (**G**) Changes in SW to BW ratio. (**H**) Changes in TC in the spleen. (**I**) Changes in TG in the spleen. An asterisk denotes *p* < 0.05. Same number of specimens was examined in each group in biochemistry. NS, not significant.

Finally, we examined the therapeutic effect of the liposomal clodronate by multiple dose administration (Figure 4A). Although our LAL-D mouse model showed growth retardation compared to controls, there were no changes in body weight between the two LAL-D mouse groups with or without liposomal clodronate administration (Figure 4B closed circle and closed triangle). Histologically, at 4 weeks of age, Oil Red O staining was positive in the liver histology of the LAL-D mouse model with no treatment (Figure 4C top center, Supplementary Figure S5). We also noticed an accumulation of Kupper cells (arrow heads) as judged by morphologically recognized strong Oil Red O staining clusters with small nuclei in this histology (Figure 4C top center). After multiple dose administration of liposomal clodronate at 50 mg/kg intraperitoneally, this cluster of Kupper cells in the LAL-D mouse models disappeared (Figure 4C, top right).

In the spleen, Oil Red O staining was similarly only positive in the LAL-D mouse model with no treatment (Figure 4C bottom left and center). When liposomal clodronate was administered at 50 mg/kg intraperitoneally to the LAL-D mouse model, the area of white pulp became negative for Oil Red O staining (Figure 4C, bottom right). Such a histological observation was consistent with the biomarkers (Figure 4D–I). In other words, the percentage of LW/BW was partially reduced by multiple administrations of liposomal clodronate (Figure 4D), while the amount of TC and TG in the liver remained unchanged (Figure 4E,F). Consistently, the percentage of SW/BW was significantly reduced in the LAL-D mouse model with liposomal clodronate administration (Figure 4G). Similarly, the amount of TC and TG in the spleen became lower in the clodronate-treated LAL-D mouse model (Figure 4H,I). In this case, liposomal clodronate attenuated an elevation of TC and TG in spleen proteins in some mice of the LAL-D mouse model without statistical significance (Supplementary Figure S6).

## 3. Discussion

In this study, we showed that a single-dose administration of liposomal clodronate to an adult LAL-D mouse model temporally eliminated macrophages in the body, with a partial reduction in splenic lipids. Similarly, a multiple-dose administration of liposomal clodronate to the LAL-D mouse model eliminated macrophages as well as splenic lipids. The impact on splenomegaly was remarkable but only partial on hepatomegaly. We found a dissociation between steatosis and a partial reduction in hepatomegaly, demonstrating that the elimination of lipids in the hepatocytes seemed absolutely essential for the treatment of steatosis in the LAL-D mouse model. We chose liposomal clodronate as the testing agent for this study, rather than a solution of clodronate, which had a lethal effect on young mice when administered intravenously at a high concentration (Figure 1B). Note that, because we only used the minimum number of experimental animals, the risk of type II error (i.e., missing a real effect because of small *n*) could remain.

In our Lipa-deficient LAL-D mouse model, we found an elimination of macrophages, as initially expected, whereas lipids in hepatocytes remained unaltered (Figure 3B and Figure 4C). The role of macrophages in Lipa-deficient animals has been examined with a genetically manipulated mouse model [20]. When the Lipa enzyme was expressed in macrophages at nearly physiological levels in a Lipa-deficient mouse model, the concentration of lipids involving CE and TG in the liver lowered significantly compared to the Lipa-deficient mouse model. Under this condition, hepatic lipids, as determined by Oil Red O positive cells in histology, remained uncleared. This observation in the liver was comparable to our results. In fact, macrophages in the liver share a minor portion of hepatic cells. Alternatively, hepatocytes predominate in the liver, playing a key role in metabolizing lipids and other biological constituents. In a hepatocyte-specific Lipa-deficient mouse model, the Lipa enzyme activity was reduced to approximately 50% of the control animal in the liver [18]. In that model, the amount of lipids involving CE was elevated, suggesting that the Lipa enzyme in the hepatocytes also metabolizes lipids efficiently. This is also consistent with our results showing that the hepatic lipids in the liposomal clodronate-treated LAL-D mouse model remained higher.

In the gene expression profile of the liver in Lipa-deficient animals, these animals usually show a skewed expression of the M2 signature, including triggering the receptor expressed on myeloid cells 2 (Trem2) and matrix metalloproteinase-12 (Mmp12) [35,36]. Thus, macrophages in the liver specialized for lipid-storage, rather than inflammation induction, play a major role in this steatosis model. In general, the role of macrophages in therapeutic contribution totally depends on the mechanism of disease and the nature of macrophages [37,38]. M1 macrophages play a key role in inflammatory settings by excreting the inflammatory cytokine tumor necrosis factor-α (TNF-α). In contrast, M2 macrophages express a variety of phagocytic receptors and anti-inflammatory cytokine transforming growth factor-β (TGF-β). Generally, the removal of macrophages during inflammatory disorders is therapeutically effective. Such examples include Concanavalin A-induced hepatitis and collagen-induced arthritis [30], and cancerous peritonitis [39].

MASLD and MASH are prevalent in developed countries [25,26]. For the experimental steatosis model, two models have been established: the methionine choline-deficient diet-induced obesity model and the high-fat diet-induced obesity model. In the methionine choline-deficient diet-induced obesity model, a clear therapeutic effect of liposomal clodronate for steatosis was reported [40,41]. In the high-fat diet-induced obesity model, clodronate treatment also reduced body weight and hepatic lipid content by intraperitoneal administration [33]. Given that the Lipa-deficient mouse model exhibits a skewed M2 macrophage gene expression profile with accumulating hepatic lipid, it is reasonable to assume that the Lipa-deficient LAL-D mouse model develops steatosis in a similar mechanism to the high-fat diet-induced obesity model.

The mechanism of clodronate on macrophage depletion involves an apoptotic effect on these cells [29,42]. Consistently, when we examined the histology of a clodronate-treated liver at 200 mg/kg, we were no longer able to identify a cluster of Kupffer cells characterized by an accumulation of cells with smaller nuclei with denser lipids in the cytosol (Figure 3B top far right and bottom far right; Figure 4C top right). Similarly, in the spleen, we were also able to easily identify the Oil Red O-negative area in the white pulp (Figure 4C, bottom right). In fact, an elimination of splenic macrophages was well correlated with a lowered splenic TC and TG (Figure 4H,I). Thus, we considered that most lipids in the spleen were stored in macrophages. In sharp contrast, the elimination of macrophages had a marginal effect on hepatic TC and TG in the liver (Figure 4E,F), suggesting that hepatocytes, but not Kupffer cells, store a large portion of hepatic lipids, while the positivity of Oil Red O staining exhibited faint red/pinkish compared to that in Kupffer cells. In clinical settings, clodronate is used for the treatment of bone disorders [43]. A combination of clodronate and enzyme replacement therapy of LAL-D is expected to reduce peripheral lipids and, thus, might show some beneficial outcome. However, a potential adverse effect on bone and tooth needs to be considered across the developmental period [44].

There are many options when preparing liposomal clodronate. It can be purchased from a commercial source as a liposomal formulation as we did [30,41]. Alternatively, a liposomal formulation could be prepared in each laboratory with a specialized formulation [34,42]. The therapeutic efficacy of a liposomal agent primarily depends on the concentration of a small molecule compound encapsulated in the liposome. The physical properties of liposomes, such as their diameter, could modulate the pharmacological properties of liposomal agents. In other words, a larger liposome in size is capable of encapsulating a higher amount of pharmacological agent, leading to an enhanced therapeutic outcome when the range of concentration is appropriate. In contrast, a smaller liposome could be beneficial for specific delivery of the agent. For example, a smaller liposome is chosen when the elimination of a tumor is designed, because the liposome needs to migrate through a narrower biological path (i.e., blood vessel) to the target [39,45,46]. Based on this background, the comparison of effective concentrations between different preparations of liposomes is occasionally difficult, particularly when the composition of lipids, method of preparation, and physical properties (such as diameter) of the final liposomal agent are not fully disclosed. In fact, our preliminary examination of intravenous administration of commercially available liposomal clodronate (averaged diameter = 300 μm) showed lethality to LAL-D mice, suggesting that the potential of drug-associated adverse effects needs to be studied in the early stages of a study (Figure 1B).

In conclusion, we demonstrated the therapeutic efficacy of liposomal clodronate in our LAL-D mouse model. The administration of liposomal clodronate lowered splenic lipids by eliminating macrophages. Kupffer cells were effectively eliminated under our experimental conditions, while hepatic lipids remained almost unaltered. We also found that the liposomal formulation was better than the clodronate solution alone, particularly when administered to young mice.

## 4. Materials and Methods

### 4.1. Reagents

Liposomal clodronate (#16001003: averaged diameter = 300 μm) was purchased from Katayama Chemical Industries (Osaka, Japan). Disodium clodronate tetrahydrate (D4160) was purchased from Tokyo Chemical Industries (Tokyo, Japan). PBS was purchased from Gibco (Tokyo, Japan). Other reagents used were the highest grade commercially available.

### 4.2. Animals

LAL-D mouse model has been described previously [35]. The mice were fed with standard chow ad libitum (CE-2, CLEA Japan, Inc., Tokyo, Japan) and were maintained on 12 h-light/dark cycles (08:00–20:00).

### 4.3. Animal Ethics

The protocol for animal experiments was approved by the Animal Committee of the National Center for Child Health and Development, Tokyo, Japan (A2018-003, approved on 26 November 2018). All methods were carried out in accordance with the relevant guidelines and regulations. The minimum number of experimental animals was used under the 3R principle (Replacement, Reduction, and Refinement).

### 4.4. Genotyping

For genotyping, genomic DNA from mouse tail clippings or tissue was isolated with 10 mg/mL Proteinase K at 56 °C for 15 h. The genome DNA was amplified using a set of the following primers: forward primer, GCTTGTCCATTCTAAACGTTACCAA; reverse primer, CCGTTGTTGCCTTTCCTCCATAG. PCR reaction was performed using a 10 μL reaction mixture containing Quick Taq^®^ HS DyeMix (Toyobo, Tokyo, Japan) at 94 °C for 2 min followed by 35 cycles of 94 °C for 30 s, 55 °C for 30 s, and 68 °C for 30 s, respectively. The amplicon was separated on 2.5% agarose gel.

### 4.5. Clodronate Administration

An aliquot of liposomal clodronate or clodronate solution in PBS was injected intraperitoneally or intravenously through the retro-orbital sinus plexus.

### 4.6. Lipid Quantification

An aliquot of tissue homogenate (50 μL) was reacted with an assay solution of either LabAssay cholesterol (#635-50981; Fujifilm Wako Pure Chemical Corporation, Osaka, Japan) for total cholesterol or LabAssay triglyceride (#632-50991; Fujifilm Wako Pure Chemical Corporation) for TG, respectively. Quantification was performed with an absorbance at 600 nm using a 96-well plate reader, Flex Station 3 (Molecular Devices, San Jose, CA, USA).

### 4.7. Oil Red O Staining

Pieces of the liver and spleen were dissected and fixed in 4% paraformaldehyde at room temperature. Next, the cryoprotected specimen was embedded in Tissue-Tek O.C.T. compound (#4583; Sakura Finetek, Tokyo, Japan). Then, a 10 μm section was prepared using a 1860UV model cryostat (Leica, Tokyo, Japan). Air-dried specimens were treated with saturated Oil Red O (#154-02072; Fujifilm Wako Pure Chemical Corporation) solution in 60% isopropyl alcohol for 5 min. Then, the nucleus was counterstained with Mayer’s hematoxylin (#131-09665, Fujifilm Wako Pure Chemical Corporation). Images were acquired using a microscope model BZ-X710 with imaging software BZ-X Viewer (Keyence, Tokyo, Japan).

### 4.8. Statistics

Statistical differences among the control, disease model, and treatment groups were analyzed using one-way analysis of variance (ANOVA). Post hoc pairwise comparisons were performed using the Tukey–Kramer test, assuming a normal distribution for each group. A *p*-value of less than 0.05 was considered statistically significant. All statistical analyses were performed in Google Colaboratory using Python (version 3.13.15) with Pandas (2.2.3), SciPy (version 1.16.3), and statsmodels (0.14.6) libraries.

## Data Availability

The original contributions presented in this study are included in the article/Supplementary Materials. Further inquiries can be directed to the corresponding author.

## Supplementary Materials

The following supporting information can be downloaded at: https://www.mdpi.com/article/10.3390/ijms27198694/s1. Refs. [17,35,36,47,48] are cited in Supplementary Materials.
