# Supplementary material for: Effect of Clodronate on the Pathogenesis in a Mouse Model of Lysosomal Acid Lipase Deficiency
Source: Int J Mol Sci. 2026 Sep 29;27(19):8694. doi: 10.3390/ijms27198694 (PMC13665179; doi:10.3390/ijms27198694)
Supplement: Supplementary file 1 [file ijms-27-08694-s001.zip › ijms-4504332-supplementary.pdf]

Supplementary Materials and Methods

Animals

LAL-D mouse model has been described previously [1]. The mice were fed with standard chow ad libitum (CE-2, CLEA Japan, Inc., Tokyo, Japan) and were maintained on 12 h-light/dark cycles (08:00–20:00).

Animal ethics

The protocol for animal experiments was approved by the Animal Committee of the National Center for Child Health and Development, Tokyo, Japan (A2018-003). All methods were carried out in accordance with the relevant guidelines and regulations.

Oil red O staining

Pieces of the liver and spleen were dissected and fixed in 4% paraformaldehyde at room temperature. Next, the cryoprotected specimen was embedded in Tissue-Tek O.C.T. compound (#4583; Sakura Finetek, Tokyo, Japan). Then, a 10 mm section was prepared using a 1860UV model cryostat (Leica, Tokyo, Japan). Air-dried specimens were treated with saturated Oil Red O (#154-02072; Fujifilm Wako Pure Chemical Corporation) solution in 60% isopropyl alcohol for 5 min. Then, the nucleus was counterstained with Mayer's hematoxylin (#131-09665, Fujifilm Wako Pure Chemical Corporation). Images were acquired using a microscope model BZ-X710 with imaging software BZ-X Viewer (Keyence, Tokyo, Japan).

Lipid quantification

An aliquot of tissue (50 mL) was reacted with an enzyme assay solution of either LabAssay cholesterol (#635-50981, Fujifilm Wako Pure Chemical Corporation, Tokyo, Japan) for total cholesterol or LabAssay triglyceride (#632-50991) for triglyceride quantification, respectively.

Statistics

Statistical differences among the control, disease model, and treatment groups were analyzed using one-way analysis of variance (ANOVA). Post hoc pairwise comparisons were performed using the Tukey–Kramer test, assuming a normal distribution for each group. A *P*-value of less than 0.05 was considered statistically significant. All statistical analyses were performed by Google Colaboratory using Python (version 3.13.15) with Pandas (2.2.3), SciPy (version 1.16.3), and statsmodels (0.14.6) libraries.

Supplementary Figure legends

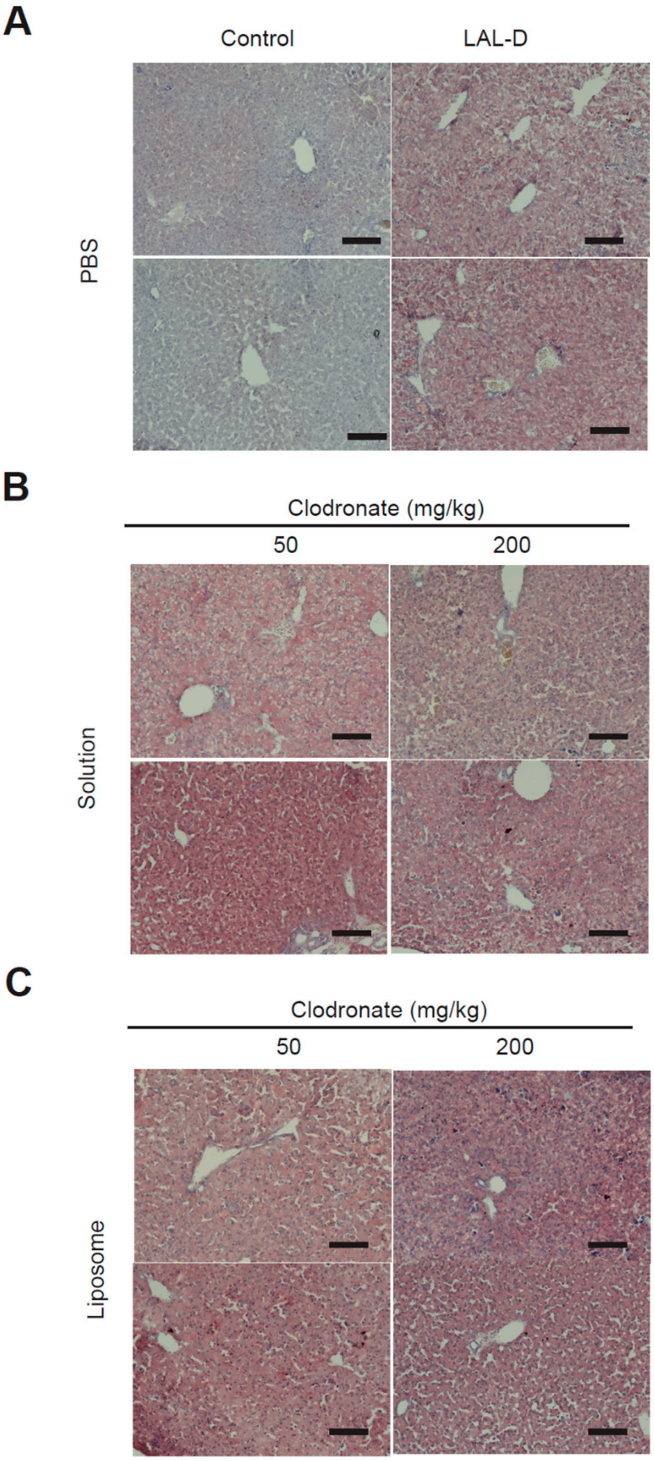

Supplementary Figure S1. Oil Red O staining of histology section of LAL-D mouse model at 2 weeks of age by single-dose administration of liposomal clodronate. (A) Oil Red O staining of liver section. Two representative images of control mice with PBS (Left, n = 4) and LAL-D mice with PBS (Right, n = 3) are shown. (B) Oil Red O staining of liver section. Two representative images of LAL-D mice with 50 mg/kg clodronate solution in PBS (Left, n = 5) and LAL-D mice with 200 mg/kg chlodronate in PBS (Right, n = 3) are shown. (C) Oil Red O staining of liver section. Two representative images of LAL-D mice with 50 mg/kg liposomal clodronate (Left, n = 4) and LAL-D mice with 200 mg/kg liposomal clodronate (Right, n = 3) are shown. Scale bar: 100 m. These mice were different from those in Fig. 2.

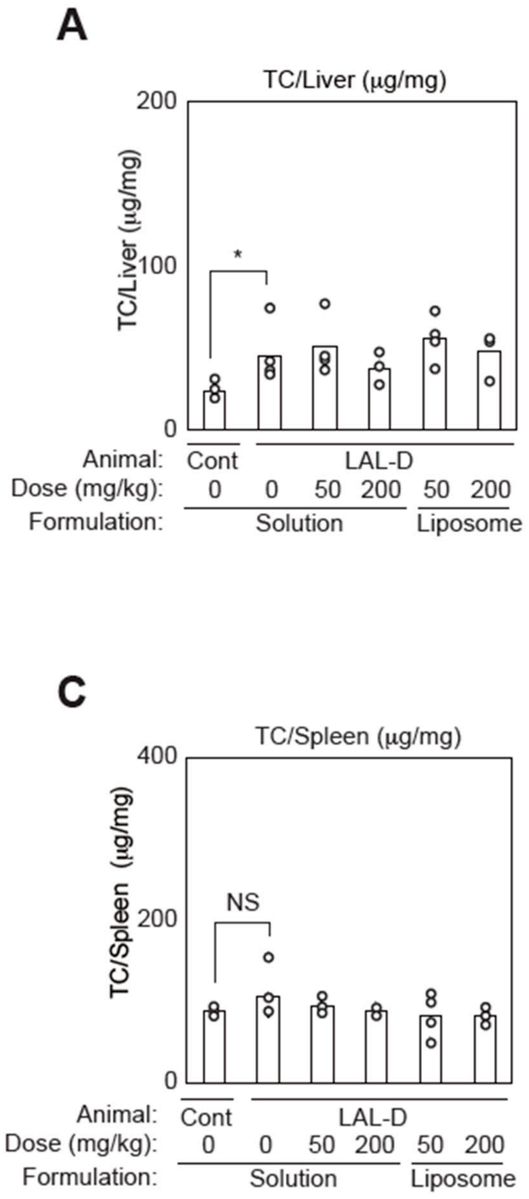

Supplementary Figure S2. Level of TC and TGs in young LAL-D mouse model at 2 weeks of age by single-dose administration of clodronate. (A) Level of TC (mg/mg protein) in liver homogenate of control or clodronate-treated LAL-D mouse model. (B) Level of TGs (mg/mg protein) in liver homogenate of control or clodronate-treated LAL-D mouse model. (C) Level of TC (mg/mg protein) in spleen homogenate of control or clodronate-treated LAL-D mouse model. (D) Level of TGs (mg/mg protein) in spleen homogenate of control or clodronate-treated LAL-D mouse model. Each group contained n = 3 - 4 animals. Each circle represents a measurement of each mouse. * indicates $P < 0.05$.

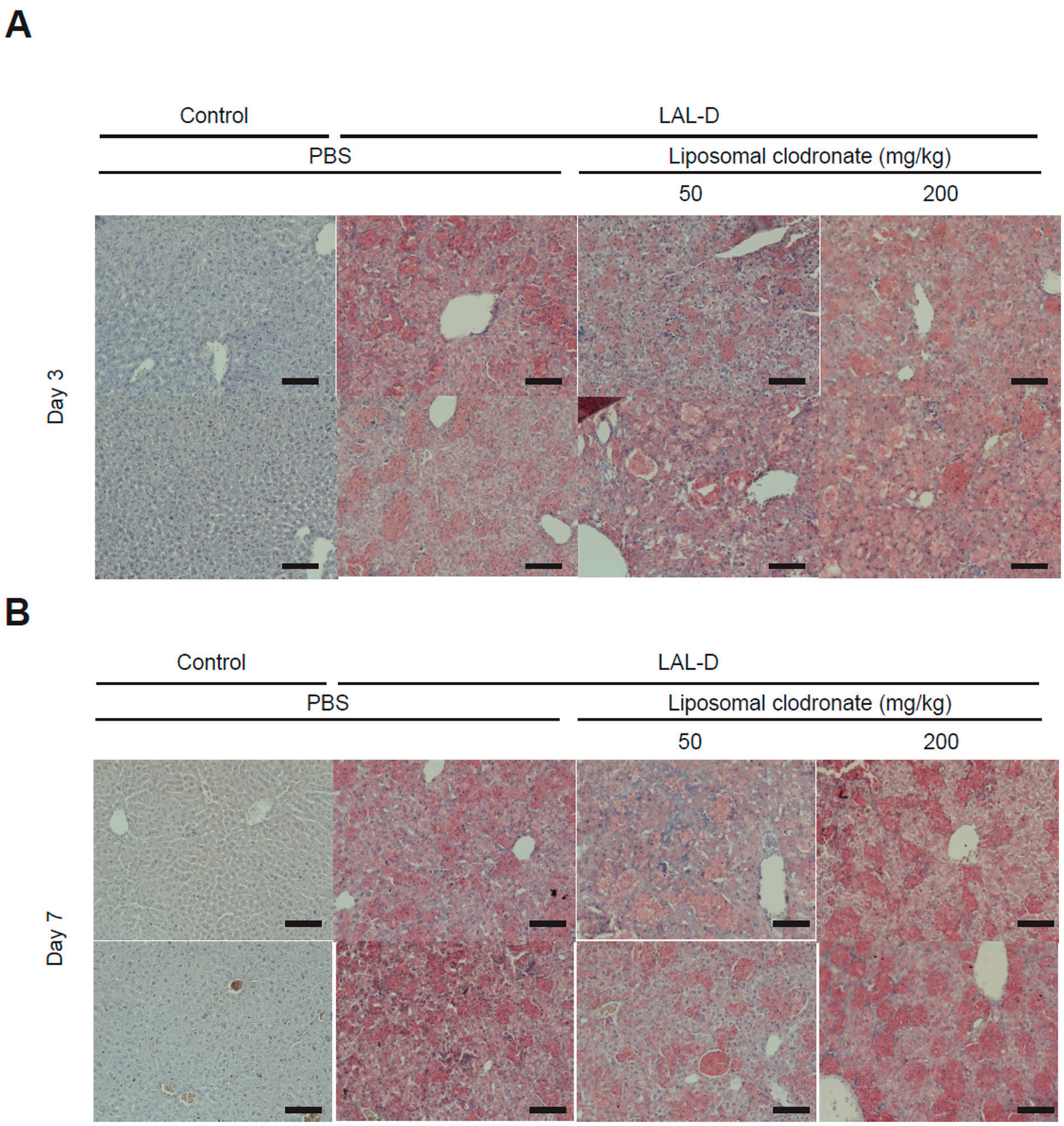

Supplementary Figure S3. Oil Red O staining of histology section of LAL-D mouse model at 8 weeks of age by single-dose administration of liposomal clodronate. (A) Oil Red O staining of liver section on Day 3. Two representative images of control mice with PBS (Far left, n = 7), LAL-D mice with PBS (Middle left, n = 4), LAL-D mice with 50 mg/kg liposomal clodronate (Middle right, n = 5), LAL-D mice with 200 mg/kg liposomal clodronate (Far right, n = 3) are shown. Scale bar: 100 m. These mice were different from those in Fig. 3. (B) Oil Red O staining of liver section on Day 7. Two representative images of control mice with PBS (Far left, n = 7), LAL-D mice with PBS (Middle left, n = 4), LAL-D mice with 50 mg/kg liposomal clodronate (Middle right, n = 7), LAL-D mice with 200 mg/kg liposomal clodronate (Far right, n = 3) are shown. Scale bar: 100 m. These mice were different from those in Fig. 3.

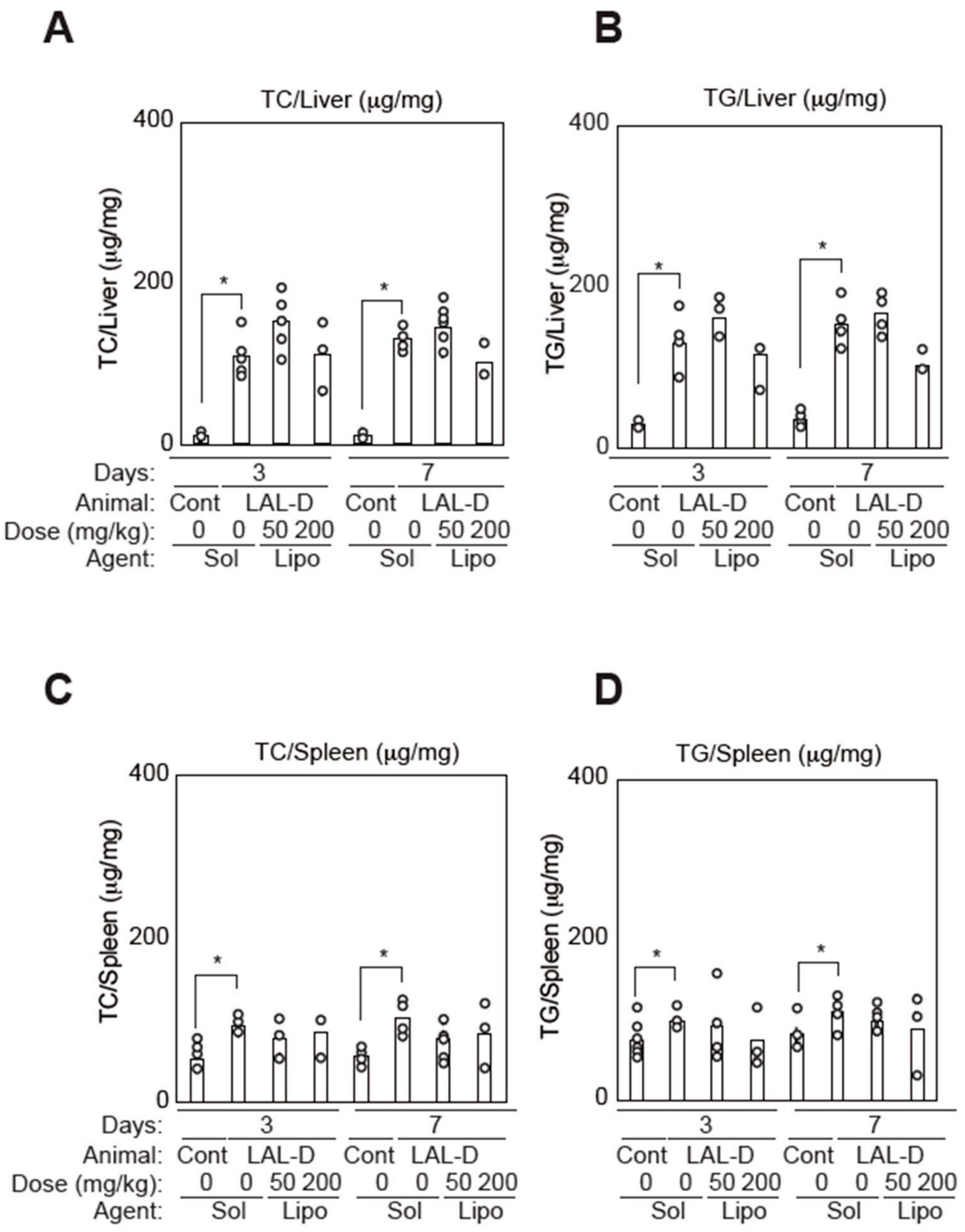

Supplementary Figure S4. Level of TC and TGs in adult LAL-D mouse model at 8 weeks of age by single-dose administration of clodronate. (A) Level of TC (mg/mg protein) in liver homogenate of control or clodronate-treated LAL-D mouse model. (B) Level of TGs (mg/mg protein) in liver homogenate of control or clodronate-treated LAL-D mouse model. (C) Level of TC (mg/mg protein) in spleen homogenate of control or clodronate-treated LAL-D mouse model. (D) Level of TGs (mg/mg protein) in spleen homogenate of control or clodronate-treated LAL-D mouse model. Each group contained n = 3 - 7 animals. Each circle represents a measurement of each mouse. * indicates $P < 0.05$.

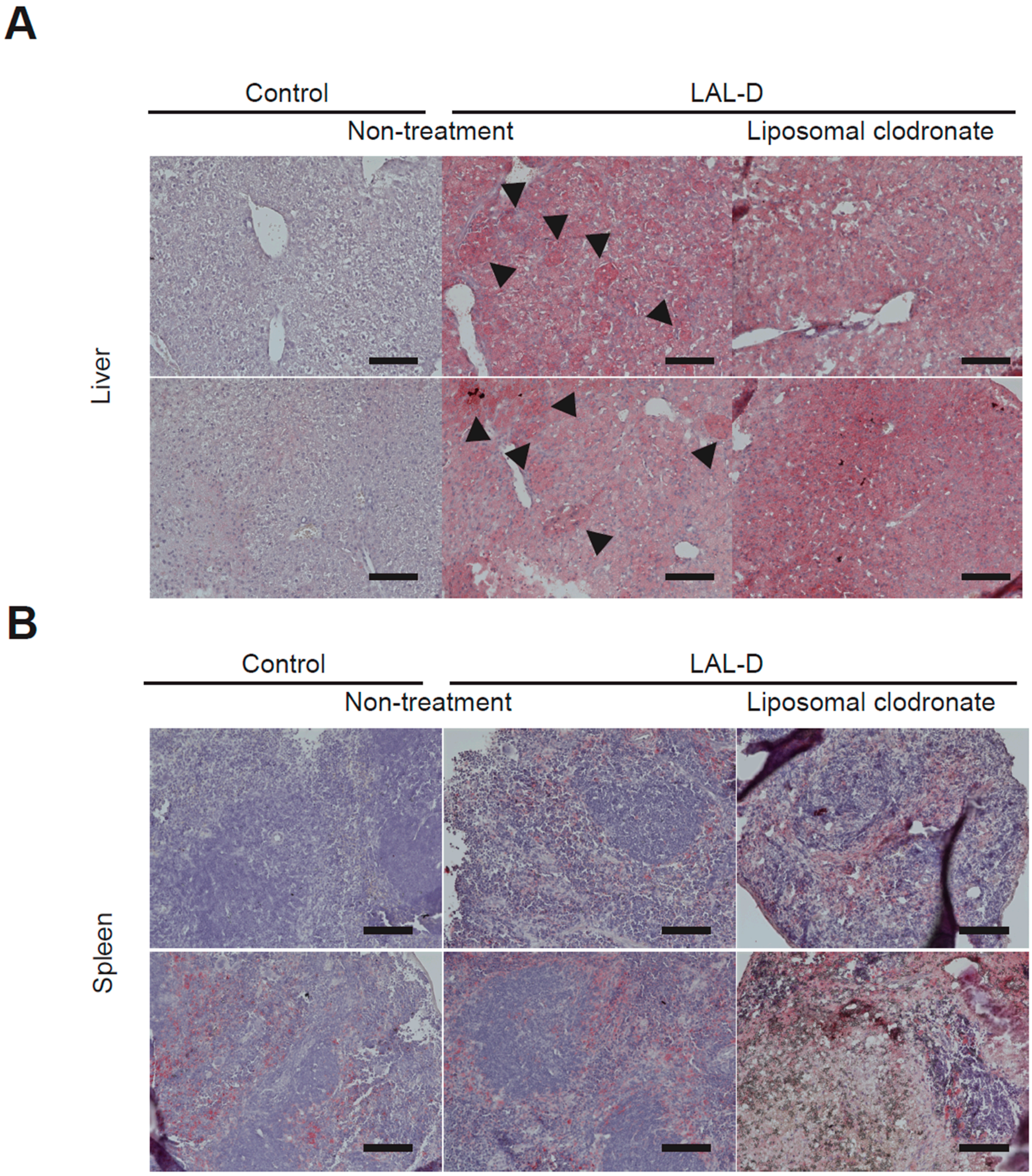

Supplementary Figure S5. Oil Red O staining of histology section of LAL-D mouse model at 4 weeks of age by multidose administration of liposomal clodronate. (A) Oil Red O staining of liver section. Two representative images of control mice with no treatment (Left, *n* = 4), LAL-D mice with no treatment (Middle, *n* = 4), LAL-D mice with 50 mg/kg liposomal clodronate (Right, *n* = 6) are shown. Arrow heads indicate Kupffer cells. Scale bar: 100 m. (B) Oil Red O staining of spleen section. Two representative images of control mice with no treatment (Left, *n* = 4), LAL-D mice with no treatment (Middle, *n* = 4), LAL-D mice with 50 mg/kg liposomal clodronate (Right, *n* = 6) are shown. Scale bar: 100 m. These mice were different from those in Fig. 4.

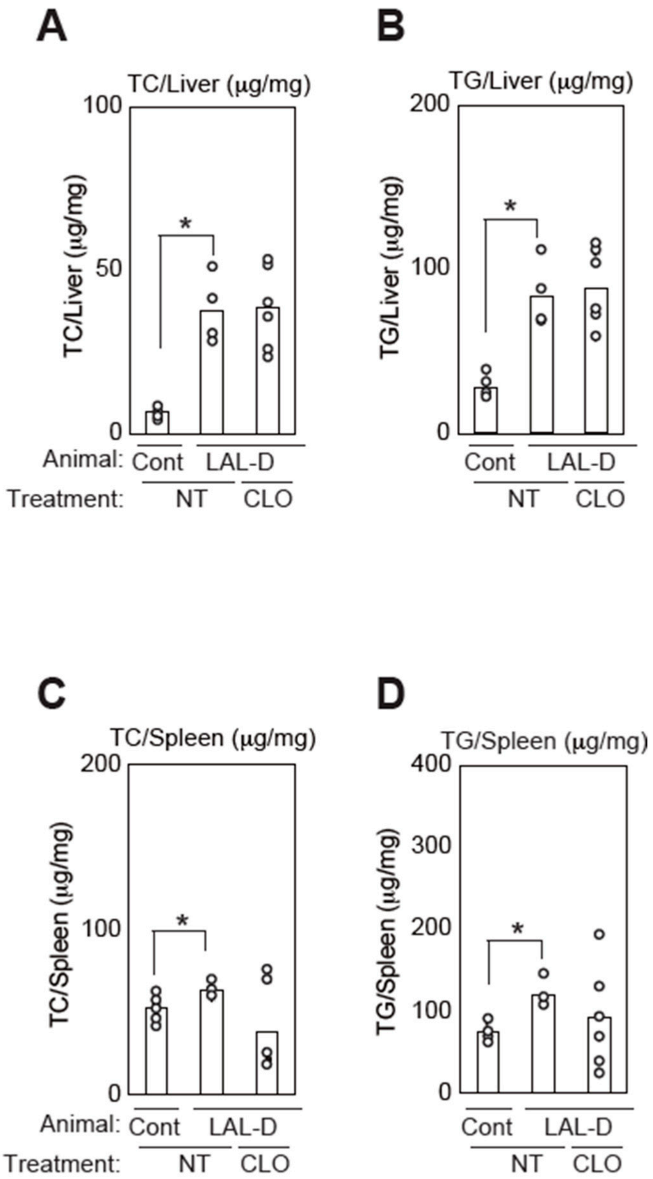

Supplementary Figure S6. Level of TC and TGs in LAL-D mouse model at 4 weeks of age by multidose administration of liposomal clodronate. (A) Level of TC (mg/mg protein) in liver homogenate of control or clodronate-treated LAL-D mouse model. (B) Level of TGs (mg/mg protein) in liver homogenate of control or clodronate-treated LAL-D mouse model. (C) Level of TC (mg/mg protein) in spleen homogenate of control or clodronate-treated LAL-D mouse model. (D) Level of TGs (mg/mg protein) in spleen homogenate of control or clodronate-treated LAL-D mouse model. Each group contained n = 3 - 6 animals. Each circle represents a measurement of each mouse. * indicates $P < 0.05$.

References

1. Mashima, R.; Takada, S.; Miyamoto, Y.; Murayama, K.; Hamazaki, T. A Novel Mouse Model of Cholesteryl Ester Storage Disorder with *Lipa* Exon 8 Splicing Junction Mutation. *Sci. Rep.* **2026**.
2. Yamazaki, N.; Ohira, M.; Takada, S.; Ohtake, A.; Onodera, M.; Nakanishi, M.; Okuyama, T.; Mashima, R. Enhanced Osteoblastic Differentiation of Parietal Bone in a Novel Murine Model of Mucopolysaccharidosis Type II. *Mol. Genet. Metab. Rep.* **2023**, *37*, 101021. https://doi.org/10.1016/j.ymgmr.2023.101021.
3. Kuentzel, K.B.; Bradić, I.; Akhmetshina, A.; Korbelius, M.; Rainer, S.; Kolb, D.; Gauster, M.; Vujić, N.; Kratky, D. Defective Lysosomal Lipolysis Causes Prenatal Lipid Accumulation and Exacerbates Immediately after Birth. *Int. J. Mol. Sci.* **2021**, *22*, 10416. https://doi.org/10.3390/ijms221910416.
4. Bianco, V.; Korbelius, M.; Vujić, N.; Akhmetshina, A.; Amor, M.; Kolb, D.; Pirchheim, A.; Bradić, I.; Kuentzel, K.B.; Buerger, M.; Schauer, S.; Phan, H.T.T.; Bulfon, D.; Hoefler, G.; Zimmermann, R.; Kratky, D. Impact of (Intestinal) LAL Deficiency on Lipid Metabolism and Macrophage Infiltration. *Mol. Metab.* **2023**, *73*, 101737. https://doi.org/10.1016/j.molmet.2023.101737.
5. Pajed, L.; Wagner, C.; Taschler, U.; Schreiber, R.; Kolleritsch, S.; Fawzy, N.; Pototschnig, I.; Schoiswohl, G.; Pusch, L.M.; Wieser, B.I.; Vesely, P.; Hoefler, G.; Eichmann, T.O.; Zimmermann, R.; Lass, A. Hepatocyte-Specific Deletion of Lysosomal Acid Lipase Leads to Cholesteryl Ester but Not Triglyceride or Retinyl Ester Accumulation. *J. Biol. Chem.* **2019**, *294*, 9118-9133. https://doi.org/10.1074/jbc.RA118.007201.
